# Medical and interventional therapy of Kasabach-Merritt phenomenon associated with Kaposiform hemangioendothelioma: A case report

**DOI:** 10.1016/j.jimed.2023.03.004

**Published:** 2023-07-31

**Authors:** Yan Zhao, Ji Cheng

**Affiliations:** a Cardiovascular Center, Tianjin Chlidren's Hospital, Tianjin, China; b Tianjin Chlidren's Hospital, 225 Machang Road, Tianjin, China

**Keywords:** Kasabach-Merritt phenomenon (KMP), Kaposiform hemangioendothelioma (KHE), Sirolimus, Interventional therapy

## Abstract

An infant with Kasabach-Merritt Phenomenon (KMP) presented with a giant subcutaneous mass in the right lower limb, severe hypofibrinogenemia, and thrombocytopenia. Glucocorticoids, along with supportive treatments including transfusion of blood products and clotting factors, were administered to reverse fatal disseminated intravascular coagulation and acute hemolysis. The glucocorticoid dose was tapered slowly, and sirolimus was added to treat the hemangiomas. The patient subsequently underwent interventional therapy. After 6 months of medical and interventional therapy, the patient was doing well with a normal platelet count, the tumor volume was markedly reduced, and the primary cutaneous lesion became pale pink. Currently, the patient remains on sirolimus, and no recurrence of thrombocytopenia or further growth of the mass was observed after six months of follow-up.

## 1 Introduction

Kasabach-Merritt phenomenon (KMP) is a life-threatening consumptive coagulopathy associated with an underlying vascular tumor. KMP is characterized by severe thrombocytopenia, microangiopathic anemia, hypofibrinogenemia, and elevated fibrin split product levels in the presence of a rapidly enlarging tumor. KMP is usually present in early infancy, and the commonly reported sites of tumors include the extremities, trunk, and neck.^1^ Uniform treatment guidelines for neonates with Kaposiform hemangioendothelioma (KHE) are lacking. Thus, many treatment modalities have been tried. Prior to 2016, no prospective studies have demonstrated the efficacy and safety of these modalities. Currently, no single treatment strategy has been consistently successful, and several different modalities are generally required.^2^ We report the case of an infant with KMP who presented with KHE on the right lower limb and his response to sirolimus and interventional therapy.

## 2 Case report

**Fig. 1:**
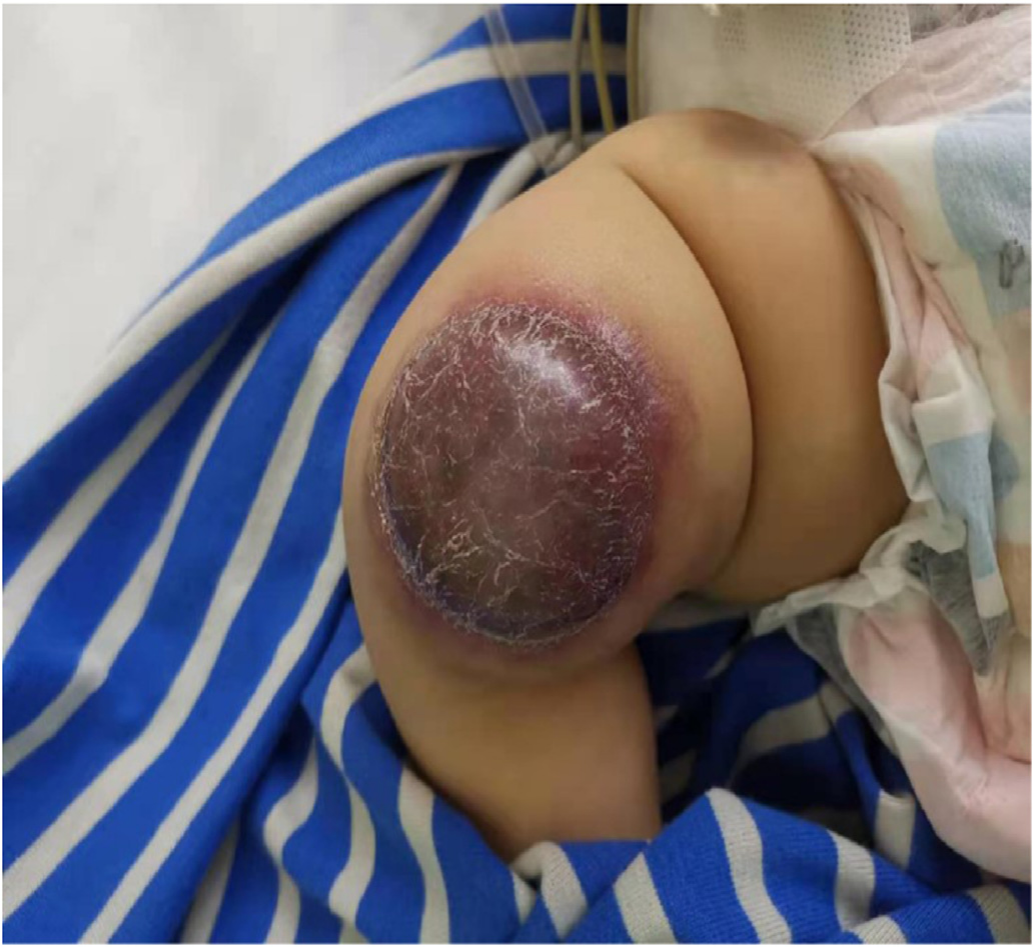
The mass on the right knee joint of the baby.

A 2-month-old male infant was admitted to our clinic with a mass in the right lower limb since birth. The mass increased in size with age and without medical treatment. The current size was approximately 5 ​× ​5 cm, the skin color was purplish red with swelling of the surrounding tissue, and the past history, personal history, and immunization were all normal. No cyanosis, convulsions, or bleeding was observed after birth. No family history of congenital malformations, tumors, or hemorrhagic disease was reported. Upon examination, the vital signs were stable. The infant was alert. Hemorrhagic spots were observed in the upper hard palate. A 5 ​× ​5 cm mass was observed at the right knee joint (Fig. 1). The mass was warm on palpation, medium in quality, and well-demarcated.

Examination of the central nervous, cardiovascular, and respiratory systems revealed unremarkable findings. The blood tests showed a moderate anemia (HGB 77 g/L) and severe thrombocytopenia (PLT 18 ​× ​109/L) through the blood routine. In addition, the coagulation function showed prolonged clotting time (PT ​> ​120 s, reference, 10.0–16.0 s; APTT 45.1 s, reference, 20.0–40.0 s; TT 31 s, reference, 14.0–21.0 s) and significantly hypofibrinogenaemia (FBG 0.541 g/L, reference, 1.8–4.0 g/L). The coagulation profile was deranged with prolonged prothrombin (international normalized ratio [INR] out of range), activated partial thromboplastin time (prolonged >120 s above control) and elevated D-dimer levels (21.29 mg/dL, reference <0.55 mg/dL). Fibrinogen levels decreased (54.1 mg/dL, reference 180–400 mg/dL). His serum electrolyte levels, along with liver function and renal function test results, were within normal limits. Electrocardiography, echocardiography, B-mode ultrasound, and chest radiography findings were all normal. No hepatosplenomegaly or mediastinal tumor was observed.

**Fig. 2:**
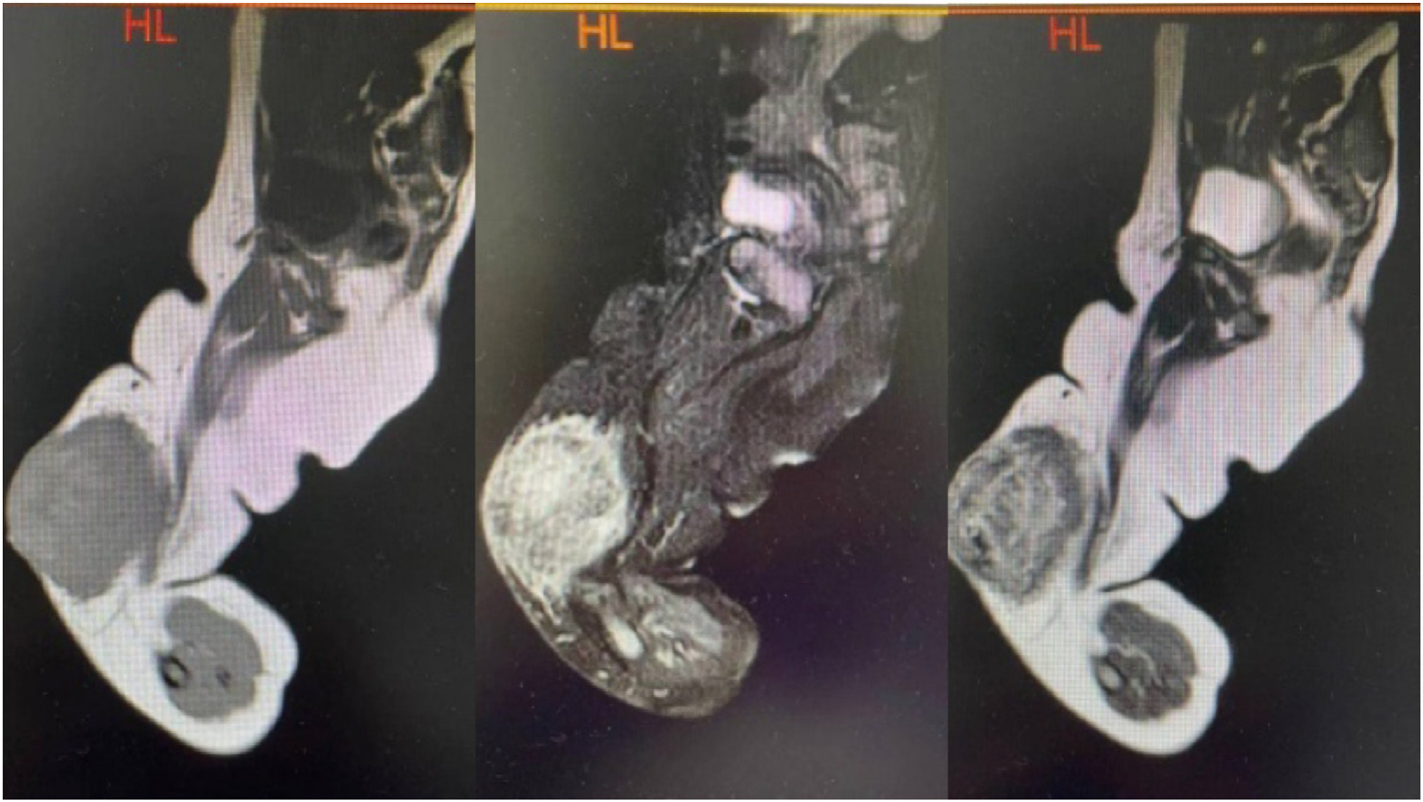
The enhanced MRI scan of the infant's right lower limb.

**Fig. 3:**
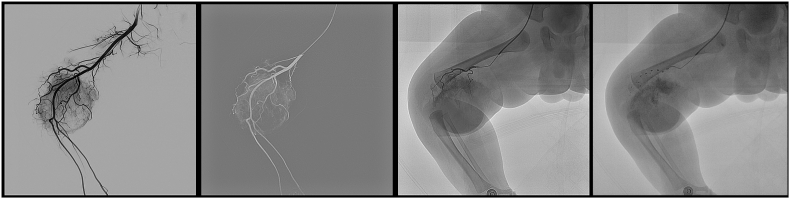
The 1.9 F microcatheter was used for superselective catheterization of the feeding artery. During the procedure, the pingyangmycin–lipiodol emulsion (PLE) was injected slowly through the catheter until the periphery of the hemangioma was completely surrounded. The PLE was made with 2 mg pingyangmycin (diluted in 2 mL of normal saline) plus 2 mL of lipiodol.

The mass showed an iso-intense signal on T1 weighted image (T1WI) and an iso-to-mild intense signal on T2 weighted image (T2WI) in the subcutaneous fat layer of the inguinal region of the right thigh. Contrast-enhanced magnetic resonance imaging (MRI) demonstrated multiple heterogeneous intense enhancement which was considered as a possibility of hemangioma (Fig. 2) (see Fig. 3).

MRI and laboratory investigations strongly suggested a diagnosis of KHE with KMP. Biopsy of the tumor was not attempted, given the potential risk for hemorrhage. The possibility of KMP was considered in view of severe thrombocytopenia, enlarging vascular lesion and coagulopathy, and oral prednisone was started (3.2 mg/kg/day) on day 1 of admission. A compassionate trial of sirolimus is warranted. The risks and benefits were explained to the family, and their consent was obtained. Conventional therapy with steroids was still ongoing and then tapered slowly, and pneumocystis prophylaxis with cotrimoxazole was additionally started owing to the potential risk of immunosuppression.

The results of the blood cell test and coagulation function went well in the first 4 days; however, on day 5, the infant had a sudden seizure. The emergency head computed tomography scan indicated no intracranial hemorrhage, and the coagulation function showed prolonged clotting time and significant hypofibrinogenemia (Table 1). Fresh frozen plasma and fibrinogen were injected to supply blood coagulation factors. The use of sirolimus and corticosteroids was continued at all times.

**Table 1:** The trending of test results during the hospitalization.

Date	Hb (g/L)	PLT (x109/L)	PT (S)	APTT (S)	PT-INR	TT (s)	FBG (g/L)	D-dimer (mg/L)
Day1	77	18	>120	45.1	ultralimit	31	0.541	21.29
Day2	65	21	11.2	33.4	0.94	18.8	1.348	49.56
Day3	73	22						
Day4	68	29						
Day5	78	54	>120	>180	ultralimit	49.9	ultralimit	8.25
Day6	73	120	10.6	29.1	0.89	19.8	1.397	12.26
Day7	72	102	9.8	26.8	0.81	18.2	1.043	34.90
Day8	79	213	11.2	26	0.94	18	1.046	11.41
Day9	75	333	11.3	27.7	0.95	19	1.381	6.65
Day10	81	385	11.3	19	0.95	17.4	1.059	5.59
Day10	Interventional embolization							
Day11	79	384	11.2	29	0.94	18.9	1.243	3.17
Day13	84	420	11.3	29.3	0.95	21.1	0.906	1.69
Day16	91	297	10.9	19.1	0.91	26.8	0.606	2.35
Day18	98	501	10.2	18.4	0.85	24.1	0.815	1.14
Day25	106	606	10.5	22.0	0.88	21.8	0.887	0.49

On day 10, the platelet count and coagulation time were normal, and the infant underwent transcatheter embolization of a Kaposiform hemangioendothelioma on the right lower limb under general anesthesia.

**Fig. 4:**
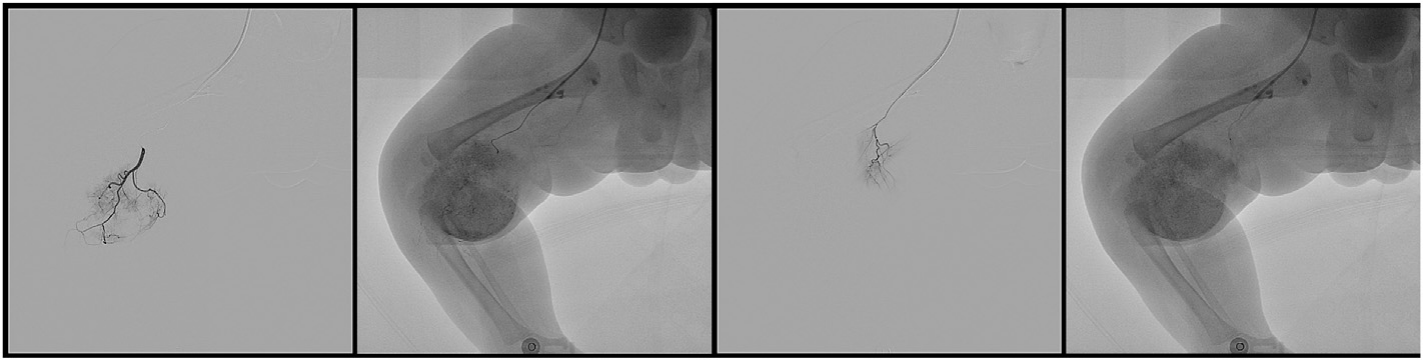
Selective right lower extremity arteriography was performed to judge the degree of embolism of the supplying arteries.

**Fig. 5:**
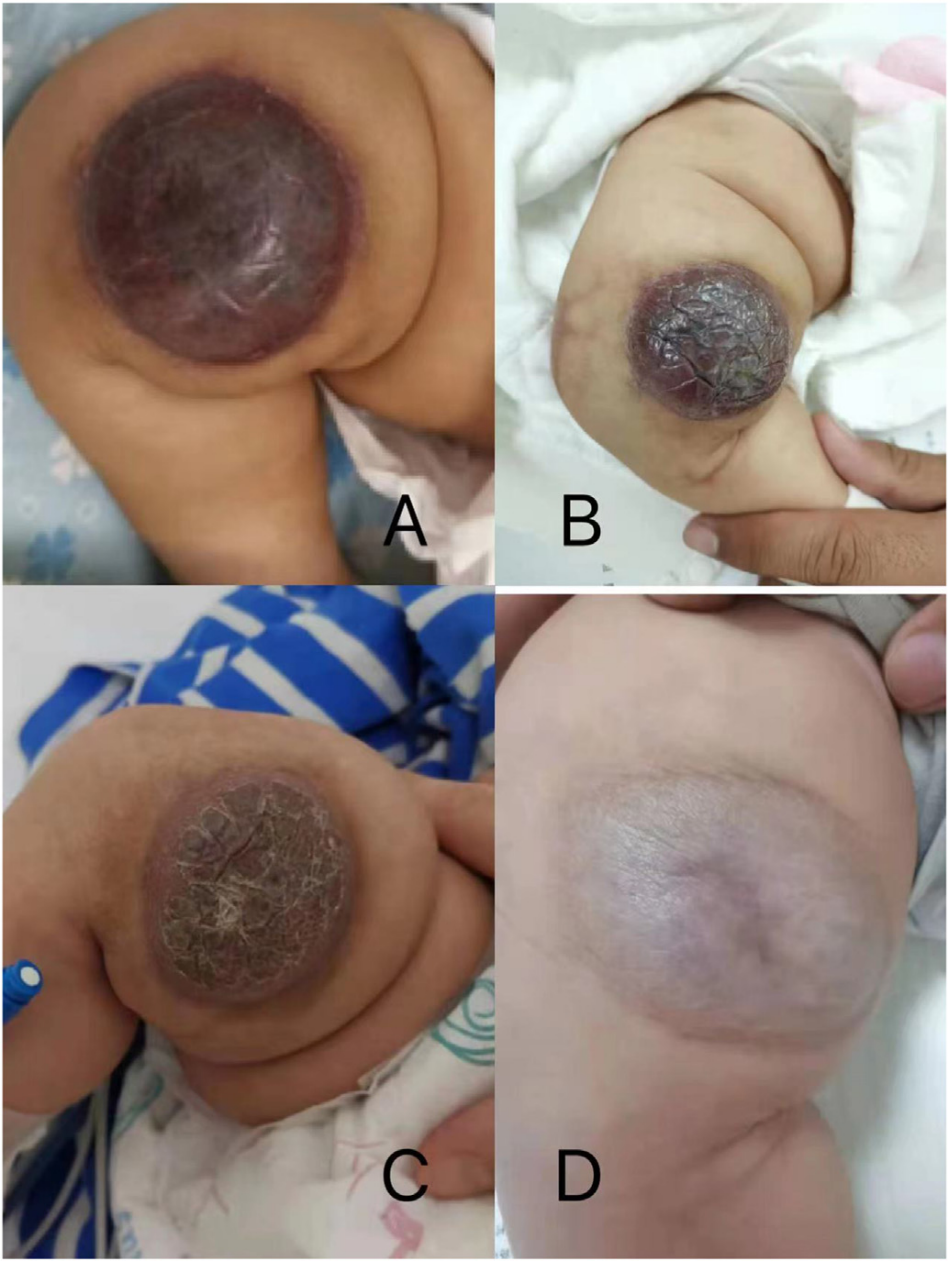
The resolution of the Kaposiform hemangioendothelioma lesion during treatment: (A) postoperative day 2; (B) after 3 weeks of treatment; (C) after 2 months of treatment; (D) after 6 months of treatment.

The patient continued to show a decrease in the size of the lesion during follow-up on sirolimus therapy at 6 months (Fig. 5) and platelet counts increased above 100 ​× ​109/L after 3 weeks of steroid and sirolimus therapy. The infant was discharged in good condition and continued on 0.8 mg/m2 of sirolimus per dose twice daily (see Fig. 4).

Follow-up 6 months after surgery indicated that the patient was in good condition, the tumor volume was significantly reduced, and the primary cutaneous lesion had become pale pink. Currently, the patient remains on sirolimus, and no recurrence of thrombocytopenia or further growth of the mass was observed after six months of follow-up.

## 3 Discussion

Kasabach and Merritt first reported a case of an infant with thrombocytopenic purpura because of what they believed to be a giant capillary hemangioma.^3^ Thereafter, the association of a capillary hemangioma and thrombocytopenia was labeled Kasabach-Merritt syndrome and this name was later changed to KMP. Kaposiform hemangioendothelioma (KHE) is a rare infiltrative vascular tumor that is typically diagnosed during infancy. KHE, first described in 1993, is characterized by a violaceous cutaneous lesion with ill-defined borders, occurring in childhood and infancy.^4^ Over 70% of patients with KHE develop the KMP, a potentially life-threatening consumptive coagulopathy. Although the incidence of KHE is low, it can cause morbidity and mortality in both children and adults. Lesions involving the neck, viscera, retroperitoneum, and mediastinum are associated with particularly high mortality rates.^5^, ^6^, ^7^, ^8^ Consequently, prompt diagnosis and appropriate management are crucial for improving the long-term prognosis of patients. Uniform guidelines for neonates with KHE are lacking. Therefore, several treatment modalities have been developed. Prior to 2016, no prospective studies had demonstrated the efficacy and safety of these modalities. Historically, KHE treatment has included high-dose steroids, chemotherapy (e.g., cyclophosphamide), antiplatelet therapy (e.g., aspirin), propranolol, embolization, radiotherapy, and sclerotherapy.^9^ Although complete surgical resection has been acknowledged as the curative gold standard for KHE, total resection is often not a viable option because of the high risk of bleeding, extent of the tumor, and/or anatomic site of the lesion.^10^ Embolization can be performed on large tumors that are unsuitable for surgical management. Interventional or percutaneous puncture was used to embolize the nutritional arteries of the tumor.^11^^,^^12^ The clinical management of high-risk patients with KMP is highly challenging. To date, no single treatment regimen has provided consistently reproducible results in terms of decreased tumor size and KMP symptom control. Should deterioration ensue during treatment, a combination therapy or an alternative treatment should be considered. However, when sirolimus is ineffective, no clinical data are available to conclusively determine the appropriate second-line management for refractory disease. Furthermore, patients with KMP who have a high probability of long-term sequelae, such as functional deformities or severe life-threatening conditions, should be treated using an aggressive strategy.^13^ We have presented a case of KHE in an infant with KMP treated with comprehensive therapy. Sirolimus may be considered as a first-line therapy or part of the treatment of KHE and KMP. However, sirolimus can take several weeks to exert its effect. Initial treatment with embolization provides rapid relief from the symptoms of KMP. Image-guided transarterial embolization may, thus, provide a valuable adjunct to sirolimus therapy in KHE/KMP.^14^

## Ethical approval

The study was approved by the ethics committee of Tianjin Children's Hospital. All clinical practices and observations were conducted in accordance with the Declaration of Helsinki.

## Patient consent

Written informed consent was obtained from the patient for publication of the case report and any accompanying images.

## Declaration of competing interest

We declare that we do not have any commercial or associative interest that represents a conflict of interest in connection with the work submitted.
